# P2P Lending Default Prediction Based on AI and Statistical Models

**DOI:** 10.3390/e24060801

**Published:** 2022-06-08

**Authors:** Po-Chang Ko, Ping-Chen Lin, Hoang-Thu Do, You-Fu Huang

**Affiliations:** 1 Department of Intelligent Commerce, National Kaohsiung University of Science and Technology, Kaohsiung 82445, Taiwan; cobol@nkust.edu.tw; 2 AI Fintech Center, National Kaohsiung University of Science and Technology, Kaohsiung 82445, Taiwan; lety@nkust.edu.tw (P.-C.L.); hyoufu@nkust.edu.tw (Y.-F.H.); 3 Department of Finance and Information, National Kaohsiung University of Science and Technology, Kaohsiung 82445, Taiwan; 4 The Faculty of E-Commerce, University of Economics, The University of Danang, Danang 550000, Vietnam

**Keywords:** P2P lending default prediction, data processing, AI model, statistical model

## Abstract

Peer-to-peer lending (P2P lending) has proliferated in recent years thanks to Fintech and big data advancements. However, P2P lending platforms are not tightly governed by relevant laws yet, as their development speed has far exceeded that of regulations. Therefore, P2P lending operations are still subject to risks. This paper proposes prediction models to mitigate the risks of default and asymmetric information on P2P lending platforms. Specifically, we designed sophisticated procedures to pre-process mass data extracted from Lending Club in 2018 Q3–2019 Q2. After that, three statistical models, namely, Logistic Regression, Bayesian Classifier, and Linear Discriminant Analysis (LDA), and five AI models, namely, Decision Tree, Random Forest, LightGBM, Artificial Neural Network (ANN), and Convolutional Neural Network (CNN), were utilized for data analysis. The loan statuses of Lending Club’s customers were rationally classified. To evaluate the models, we adopted the confusion matrix series of metrics, AUC-ROC curve, Kolmogorov–Smirnov chart (KS), and Student’s t-test. Empirical studies show that LightGBM produces the best performance and is 2.91% more accurate than the other models, resulting in a revenue improvement of nearly USD 24 million for Lending Club. Student’s t-test proves that the differences between models are statistically significant.

## 1. Introduction

Crowdlending or P2P lending has gradually become more popular worldwide due to the accelerated growth of financial technologies. In P2P lending, “users of the platform are lending capital directly to their peers, mediated by a platform without a bank standing in between” [1]. Therefore, both borrowers and lenders can trade without the intervention of financial institutions. Aside from the convenience of credit review and loan processing, crowdlending services are also cost-effective. P2P platforms are more advantageous for borrowers with economic difficulties or low credit scores than traditional financial institutions because they can easily match borrowers with lenders. In addition, individual investors who are willing to tolerate the extra risk may receive a higher return; thus, the benefits of P2P lending extend to both parties. Crowdlending has the distinct advantage of not requiring investors to lend all of their money to the same person; instead, they can diversify the investment amount to different borrowers to decrease risks.

From another perspective, however, if P2P platforms fail to formulate relevant regulations and supervision, they can only rely on ethical standards. For instance, people with higher education are more likely to obtain loans and pay them back [2]. Taking China as an example, despite the rapid development of crowdlending in this country, the Chinese market faces a high moral hazard and negative consequences, including numerous platform failures and loss of investors’ deposits. The business continuity of P2P platforms and the protection of investors and lenders are therefore significant concerns. As of early 2018, approximately 6000 P2P platforms had been registered in China, generating around USD 800 billion in loans. However, a phenomenally high failure rate has accompanied this extraordinary growth. The number of P2P platforms that had ceased to operate by early 2018 amounted to over 60%. Because of ineffective regulations, many Chinese platforms went bankrupt in June 2018. There were 2834 active platforms at that time, but the outstanding loans amounted to RMB 1.317 trillion (~USD 200 billion), most of which were short-term loans. This led to a phenomenon known as runoff, where platform owners disappeared with investor funds. Due to stricter national regulations on P2P lending platforms from 2018 to 2020, only 29 platforms were still active by June 2020 [3,4].

In the United States (USA) context, policymakers provide little support for P2P lending; however, banks and P2P companies have collaborated over time. The bank–P2P lender partnership announced in April 2015 between Citi and Lending Club (LC), for example, would provide extra funding to small and medium-sized enterprises, thus legitimizing P2P lending. In 2007, LC first launched and became the largest P2P lending platform in the USA. LC and another leading platform—Prosper—currently account for about 98% of the USA market share [5]. LC only provides services to borrowers with a FICO score of 660 or higher, and its interest rate is lower than the interest on a credit card due. Therefore, LC’s users are not just those plagued by financial distress; 77.14% of borrowers use the platform to repay credit card bills. LC is also eroding the core competitive advantages of traditional banks by offering attractive interest. As a result, the demand for such personal credit loans increases daily, which means that default risk is rising.

The current situation of the P2P lending markets in two major countries, China and the USA, raises the question: What is the most optimal model to minimize the default risk of P2P lending platforms based on data provided by the borrowers? In response to this question, this paper proposes classification models to control the risk of default and ensure the sustainability of P2P platforms. Based on public information from LC, we present methodologies for processing this company’s dataset. Good data are essential to building good models in financial technology. Data are now comparable to gold or currency, and, in the future, they may be considered an intangible asset [6]. Besides collecting the original data, pre-processing plays a major role in converting data into decision-making information. The first objective of this research (1) is to propose a series of methods to improve data quality and construct models. Raw data went through feature screening, data conversion, 10-fold cross-validation, and dataset balancing steps. The second objective (2) is to apply three statistical models, namely, Logistic Regression, Bayesian Classifier, and LDA, and five AI models, namely, Decision Tree, Random Forest, LightGBM, ANN, and CNN, for data analysis. This study examined the performance of each model in P2P lending default prediction under similar data structures. The last purpose of this research (3) is to propose metrics to evaluate prediction models. Once the model results are obtained, if we only analyze their accuracy to measure the model’s pros and cons, there might be blind spots, eventually leading to decision-making bias. Therefore, the authors also used other evaluation methods, including the confusion matrix series of metrics, AUC-ROC curve, and Kolmogorov–Smirnov chart (KS). Lastly, Student’s t-test was applied to determine whether the performance of the best model is significantly better than its next-ranked models.

P2P platforms face various risks, including default risk, operational risk, legal risk, liquidity risk, and information asymmetry. This research mainly aims to improve P2P default risk and information asymmetry. The former is the risk that a lender bears if the borrower does not meet their obligations. Since P2P lending is unsecured, the platform has difficulty grasping the borrower’s other assets, resulting in a low recovery rate. The latter is the risk that borrowers and lenders lack information about one another’s situation; thus, it is impossible to accurately judge the other party’s credit level. Information asymmetry results in excessive reliance on credit rating systems of the platform [7]. This study aimed to build prediction models so that investors can obtain borrowers’ credit levels early, increase information transparency, and reduce default risk and information asymmetry. Investors can also handle risky debts in advance to reduce losses by selling them.

Our study contributes to the literature review of this topic by covering and comparing the most recent advanced AI and statistical models and nearly all of the appropriate evaluation methods, both theoretically and experimentally. In addition, we also identify and fix weaknesses in previous studies concerning variable selection and data processing.

## 2. Literature Review

Researchers have previously developed many statistical and machine learning models to predict the default risk of P2P lending platforms, and studies based on the LC dataset account for the majority. Therefore, it can be said that LC data are a benchmark dataset in P2P lending.

### 2.1. Studies Using LC Dataset

Petr Teply et al. [8] exploited the LC dataset from 2009 to 2013 to build a ranking score system among ten classifiers, including Logistic Regression, ANN, LDA, Linear SVM, Random Forest, Bayesian net, radio SVM, Naïve Bayes, classification and regression tree, and k-Nearest Neighbor. The results showed that Logistic Regression was ranked number 1 based on the Percentage Correctly Classified measurement. In the study of Milad Malekipirbazari et al. [9], Random Forest was more effective in evaluating LC’s risk than Logistic Regression and FICO credit scores. Sriharsha Reddy [10] applied the Boosted Tree classification method to the LC dataset to predict the probability of delinquency. The findings revealed that XGboost had the best performance, with 99% accuracy for both the training set and test set. LightGBM and XGboost were used to predict the default risk of LC in the paper of Xiaojun Ma et al. [11]. The results prove that LightGBM and XGboost are both effective methods; however, the running time of LightGBM is much faster than XGboost, and the difference is more than ten times.

Duan Jing [12] used a three-hidden-layer neural network trained by the back-propagation algorithm to test the LC dataset in the 2007–2015 period. Loans were classified into safe debt, risky debt, and bad debt. Because most of the debts in the dataset are safe, the author applied the Synthetic Minority Oversampling Technique to improve the model accuracy and achieve a rate of 93%. Golnoosh Babaei et al. [13] designed a data-driven investment decision-making framework by adopting ANN and Logistic Regression to estimate the internal rate of return and the chance of default of each loan in the LC dataset. In the study of Ji-Yoon Kim et al. [14], a deep dense convolutional network was proposed for LC default prediction. This method obtained an accuracy of 79.6% by checking the flow of loan information through dense connectivity and automatically deriving discriminative features by convolution and pooling operations.

Van-Sang Ha et al. [15] built classification models using LDA based on feature selection combined with restricted Boltzmann machines (RBMs) for credit scoring tasks. The model was tested on Australian credit, German credit, and LC datasets. After the feature selection process, 12 features were chosen for the Australian credit dataset, 22 were selected for the German credit dataset, and the number for the LC dataset was 80, respectively. The accuracy rates were 86.09%, 76.7%, and 81.53%. In their study, Luis Eduardo Boiko Ferreira et al. [16] examined the creditworthiness of LC borrowers in the period from 2007 to 2016. The authors combined ensemble, cost-sensitive, and sampling methods with Logistic Regression, Decision Tree, and Bayesian learning models. Overall, the sampling techniques surpassed ensemble and cost-sensitive approaches. Yufei Xia [17] combined outlier detection techniques with a gradient boosting decision tree algorithm to establish a credit scoring model for LC and We.com datasets, effectively reducing information asymmetry.

### 2.2. Studies Using Other Datasets

In their study, Yuejin Zhang et al. [18] used Logistic Regression to determine whether borrowers will ultimately be able to get loans via Paipaidai—the largest P2P platform in China. Their findings indicate that the annual interest rate, repayment period, description, credit grade, the number of successful loans, the number of failed loans, gender, and credit score are significant factors for a successful loan. An accuracy rate of 94% was achieved. Using Naïve Bayes and data from both core credit and social network information on the Prosper platform, Radha Vedal et al. [19] categorized good and bad borrowers with an average accuracy rate of 82%. Binjie Luo et al. [20] conducted interesting research to examine the impact of herd behavior on the P2P lending market. They used Decision Tree to test the Prosper dataset. Investors can place bids on their friend’s listing through Prosper’s social network service. This study showed that investors are more inclined to follow herd behavior if they strongly prefer public information and the cost of acquiring and studying extra information. Moreover, herding on listings with more bids hurts investors.

It is noticeable that some studies on the P2P lending topic produce relatively high results; however, we also find a few problems. Included in the models of these studies were post-event variables, which are created after the loan request is approved, such as “total payment” (total_pymnt), “the month in which the loan was funded” (month_iss), or “last month payment was received” (last_pymnt). These variables severely affect the prediction outcome of classification models, as post-event variables usually give higher results (e.g., a person who has paid off all of their debts is naturally classified as risk-free). Moreover, post-event variables have no meaning in assisting investors in decision making because lenders must determine whom to lend their money to before a debt is established.

In addition, most of the datasets of P2P lending companies are often imbalanced, in which the number of safe debts is much higher than the number of risky debts. However, many studies have not addressed this issue in their classification problems, which leads to biased results. For example, a naïve classifier may focus on learning the characteristics of safe debts only and neglect the bad debts, which is, in fact, what we should really pay attention to.

Our study fills these gaps by eliminating all post-event variables and also considering the problem of data imbalance by adopting the under-sampling technique.

## 3. Research Process

**Figure 1:**
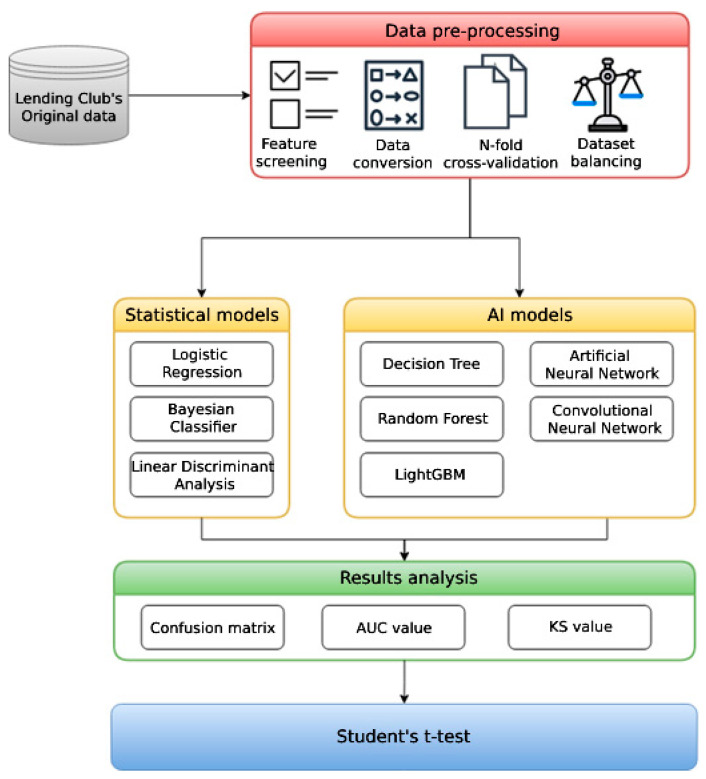
Research flow chart.

The research process in this study is divided into three main steps: data pre-processing, model construction, and results analysis. In order to increase the robustness of the models, we first observed the dataset, removed empty data, screen features, and converse data types, and used 10-fold cross-validation to divide the dataset into training sets and test sets randomly; the data imbalance issue was also taken into consideration. Our next step was to construct three statistical models and five AI models, which we evaluated by the confusion matrix series of metrics, AUC-ROC curve, Kolmogorov–Smirnov chart (KS), and Student’s t-test. The research flow chart is shown in Figure 1.

## 4. Research Data

**Figure 2:**
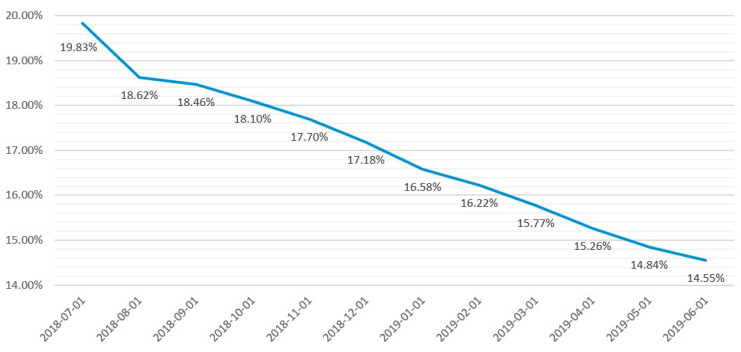
LC default rates from 2018-07 to 2019-06.

We utilized public information from Lending Club—the largest P2P platform in the USA—and examined data from 2018 Q3 to 2019 Q2. Figure 2 shows the trend of LC’s default rates from 2018-07 to 2019-06. We can observe that LC’s default rates have declined over the years; however, they are still higher than 14%.

### 4.1. Feature Screening

This paper only studies loan results after expiration. Therefore, two types of loans, “Fully-Paid” and “Charged-Off,” were selected from the original dataset. The term “Fully-Paid” indicates that a loan has been fully repaid when due, and “Charged-Off” indicates a bad debt. Other statuses of loans that are generated when the loan is not due were excluded. When feeding data into the models, “Fully-Paid” loans were labeled as 0, and “Charged-Off” loans were labeled as 1. Table 1 shows how loans are classified.

In order to reduce the burden of computational cost, more than 50% of the features with empty values, those that provided little information, and those that were irrelevant to the experiment were deleted. In addition, this research aimed to determine whether lenders should provide loans to borrowers; therefore, features that are created after the loan decision, such as “total repayment” (total_pymnt), were also excluded. After this process, 63 features remained. A detailed description of these features is given in Appendix A.

### 4.2. Data Conversion

Because features differ in their underlying nature and significance, this paper converts those features based on their Scales of Measurement to meet the data input requirements of the models.

- Nominal data: On a nominal scale, events or objects are divided into distinct categories without considering order or ranking. Instead, these categories are given unique labels, and calculations for adding, subtracting, multiplying, and dividing the nominal scales are meaningless. Therefore, this study converts nominal-scale variables into the quantitative scale to feed into the model.
- Ordinal data: The ordinal scale represents the hierarchical relationship among levels on a scale. However, it cannot measure the distance between different levels. This paper converts ordinal data such as debt level, duration of the loan, etc., into numerical data.
- Numerical data: Most of the data in this research are discrete and not highly correlated; i.e., the degree of mutual influence between customers is low. Therefore, missing data are replaced by the average values. After that, data are scaled in the range 0–1 using min–max normalization as follows:
  (1) $$x_{i}=\frac{x_{i}-x_{min}}{x_{max}-x_{min}}$$
  where
  $x_{i}$ is the normalized value;
  $x_{min}$ is the minimum value of the series to be normalized;
  $x_{max}$ is the maximum value of the series to be normalized.

This method eliminates the problem of inconsistencies in the range of variables, allowing algorithms to extract features and improve the accuracy of the models.

### 4.3. Ten-Fold Cross-Validation

**Figure 3:**
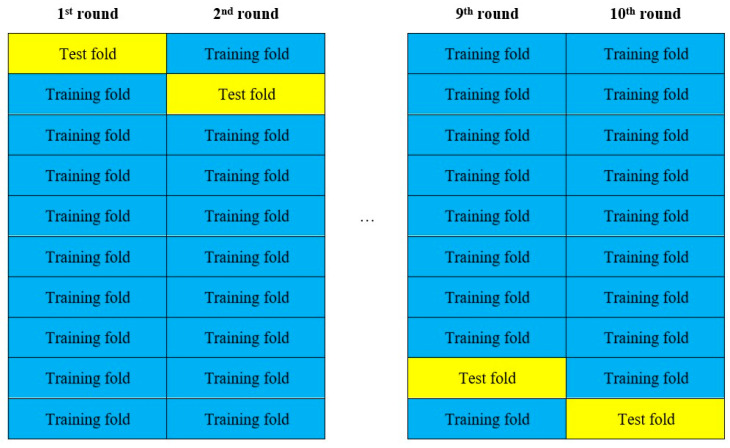
Schematic diagram of cross-validation.

Unlike time series data, the data used for this study have little correlation; that is, the behavior of an individual borrower is not highly related to the behavior of others. Therefore, we used cross-validation to resample the dataset, and 10-fold stratified cross-validation is proved to be the best method for real-world datasets [21]. Using the random grouping method, we divided the LC dataset into 10 groups (9 training sets and 1 test set) in order to prevent the occurrence of overfitting while improving the reliability and validity of the models, as shown in Figure 3.

The average value of the 10-group results was calculated to estimate the model’s performance.

### 4.4. Data Balancing

The ratio between “Fully-Paid” and “Charged-Off” loans in our dataset is about 85:15, resulting in the problem of imbalanced data (also known as data skewness). Thus, we used the undersampling technique to balance the dataset by maintaining all of the data in the “Charged-Off” class and decreasing the size of the “Fully-Paid” class to a ratio of 1:1. This process was adopted for the ten datasets divided in the previous step.

## 5. Model Construction

### 5.1. Statistical Models

Statistical models use mathematics and statistics to classify and predict data. Based on a previous article [8], this study employed three statistical models: Logistic Regression, Bayesian Classifier, and LDA. These three classic and straightforward models work quickly and do not require too much training. Despite their simplicity, these models prove to be effective in classification tasks.

#### 5.1.1. Logistic Regression

Logistic Regression is a learning algorithm used to minimize the error between its predictions and the training data in a supervised learning problem. The basic assumptions and regression equations of Logistic Regression are shown in Equation (2).

(2) $${\hat{y}}_{i}=\;P(y_{i}=1|x_{i}),\;\mathrm{where}\;0\;\leq {\hat{y}}_{i}\leq 1$$

where

$x_{i}$ is the input feature vector of the *i*th instance;
$y_{i}$ is the actual observation of the *i*th instance, $y_{i}\in 0,1$ ($y_{i}=1$ indicates a default event; $y_{i}=0\;$indicates a non-default event);
${\hat{y}}_{i}$ is the probability of default of the *i*th instance, given$\;x_{i}$;
${\hat{y}}_{i}$ follows the Sigmoid function in Equation (3):
  (3) $${\hat{y}}_{i}=S\left(z_{i}\right)=\frac{1}{1+e^{-z_{i}}}\;$$
  where $z_{i}=\alpha +\beta x_{i}\;(\alpha$ is the constant term; β is the regression coefficient).

**Figure 4:**
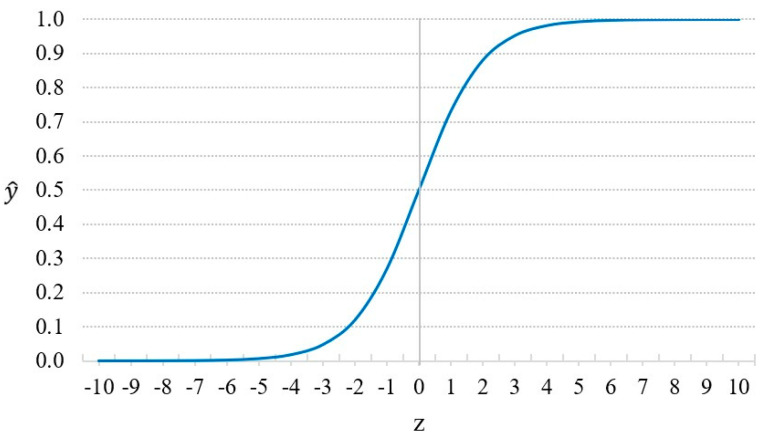
Sigmoid function.

If ${\hat{y}}_{i}>0.5$, we predict $y_{i}=1$; otherwise, we predict $y_{i}=0$. Figure 4 demonstrates the Sigmoid function.

#### 5.1.2. Bayesian Classifier

The Bayesian Classifier is a simple probability classifier based on Bayes’ theorem, which is represented as the following function.

(4) $$P(C\;|\;F_{1},\;F_{2},\ldots ,\;F_{k})=\frac{P\left(C\right)P(F_{1},\;F_{2},\ldots ,\;F_{k}\;|\;C)}{P\left(F_{1},\;F_{2},\ldots ,\;F_{k}\right)}$$

where

*C* represents the target (“Fully-Paid” or “Charged-Off” loan);
$F_{1},\;F_{2},\ldots ,\;F_{k}\;$ represent k features.

In the Bayesian Classifier, features are assumed to be independent of each other with no correlation. However, this is not the case in real life, so this model is sometimes referred to as “naïve”.

#### 5.1.3. Linear Discriminant Analysis (LDA)

R. Fisher initially proposed LDA in 1936 as an analysis method to identify different kinds of flowers [22]. LDA focuses on finding a projection hyperplane that maximizes the ratio of the between-class variance to the within-class variance to ensure optimal separability. LDA is very similar to Principal Component Analysis (PCA): both methods find the linear combination of features that best explain the data. However, PCA is a type of unsupervised learning, which does not consider the label, while LDA is a supervised learning algorithm that can be used for dimensionality reduction and classification problems.

**Figure 5:**
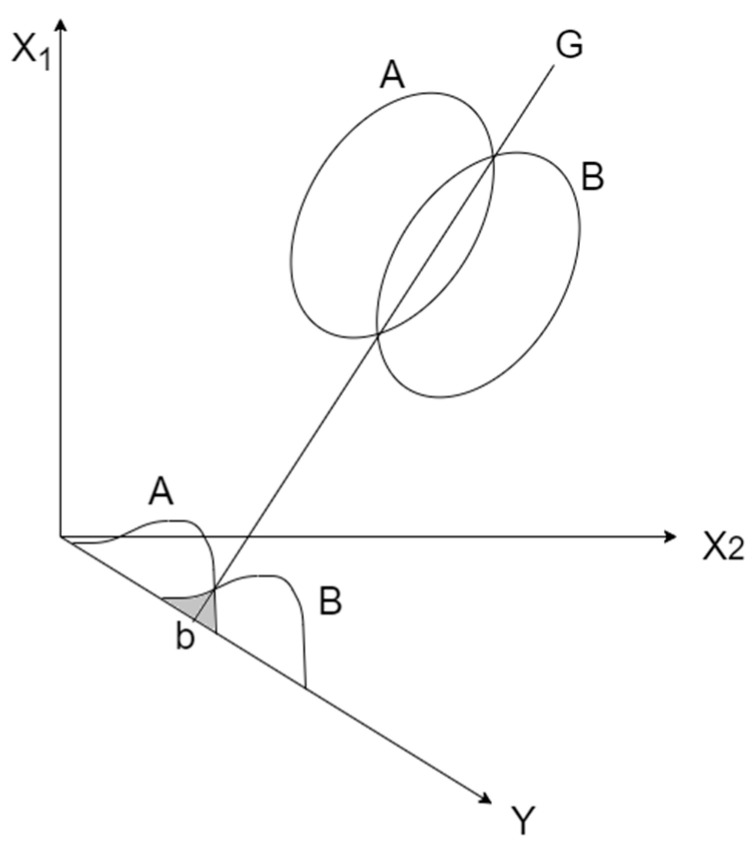
LDA classification.

For two-dimensional data, LDA is illustrated in Figure 5. There are two data classes, A and B, and then all of the data points of the two classes are projected onto a straight line Y, which is perpendicular to the straight line G generated by the intersection of the two data classes. The intersection of lines Y and G is b. The projected result on the straight line Y provides the frequency distribution of the A and B classes. At this point, the optimal ratio of the between-class variance to the within-class variance will be higher than that of other projections. Point b is called the critical point, where line Y is divided into two parts to complete the construction of the two-class model.

### 5.2. AI Models

Artificial Intelligence (AI) models are used for prediction and classification purposes by training data and analyzing features. This study adopted Decision Tree, Random Forest, LightGBM, ANN, and CNN to predict the default risk of P2P lending.

#### 5.2.1. Decision Tree

**Figure 6:**
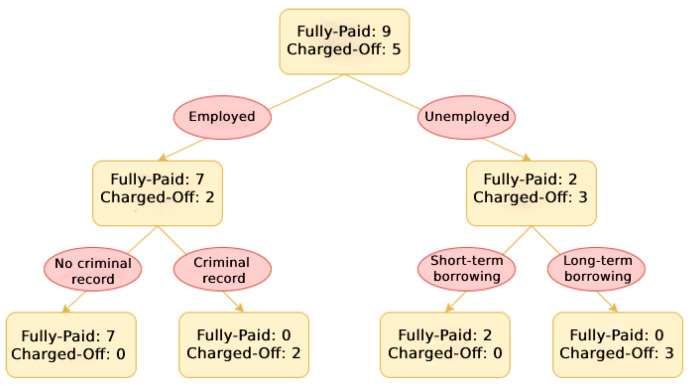
Schematic illustration of Decision Tree.

A Decision Tree is a supervised learning technique that uses a tree-based model. Internal nodes represent tests over an attribute, branches represent the test results, and leaf nodes represent the labels. An example of a Decision Tree for default risk prediction is shown in Figure 6.

The Decision Tree has some advantages, such as being easy to understand and interpret; however, it also bears a common disadvantage of overfitting if an overly complex tree is created.

#### 5.2.2. Random Forest

**Figure 7:**
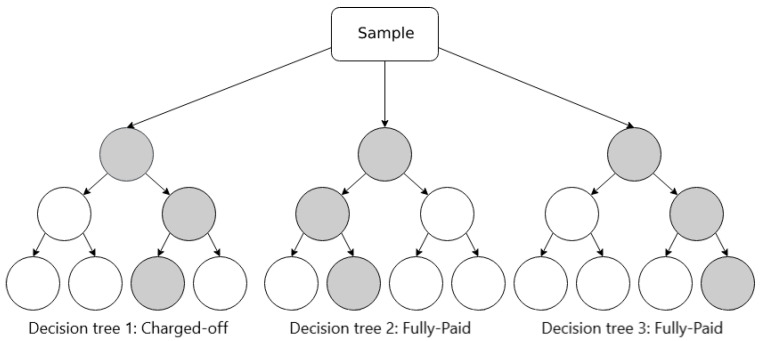
Schematic illustration of Random Forest.

In Random Forest, data samples are randomly selected to create decision trees, and these trees are merged together so that they can make better predictions. When dealing with a regression problem, we can average out the predictions of each tree (mean); when dealing with a classification problem, we can take the majority of the classes that each tree voted for (mode). Figure 7 illustrates an example of Random Forest.

#### 5.2.3. LightGBM

Gradient Boosting Decision Tree (GBDT), which was developed from Decision Trees, Random Forests, and other methods, has been widely used in recent years. Among them, Light Gradient Boosting Machine (LightGBM) shows excellent performance in classification prediction.

**Figure 8:**
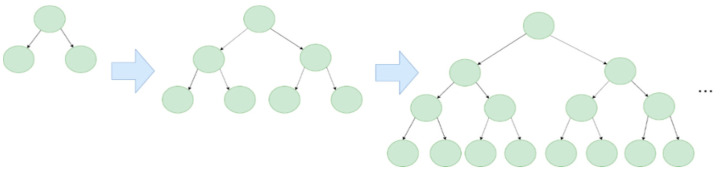
Level-wise learning tree.

**Figure 9:**
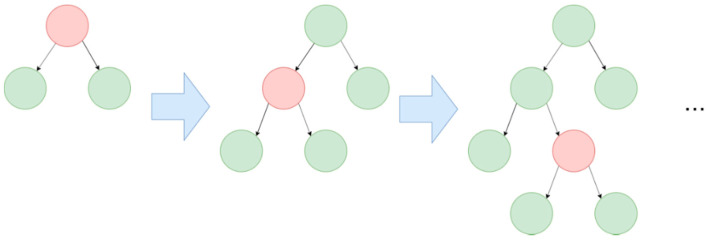
Leaf-wise learning tree.

The Gradient Boosting algorithm uses decision trees as its weak learners (week learners are classifiers that produce predictions that are just a bit better than random guessing). Each model in Gradient Boosting is learned sequentially and based on the prediction error of the previous one. GBDT has some conventional implementations, such as XGBoost and pGBRT; however, these methods have high computational costs and are therefore time-consuming. LightGBM, which Microsoft developed, is a lightweight framework for implementing GBDT. This algorithm has advantages, such as faster training speed, higher accuracy, and large-scale data handling. Unlike traditional level-wise algorithms, LightGBM grows leaf-wise. The comparison of the two methods is shown in Figure 8 and Figure 9 as follows:

#### 5.2.4. Artificial Neural Network (ANN)

**Figure 10:**
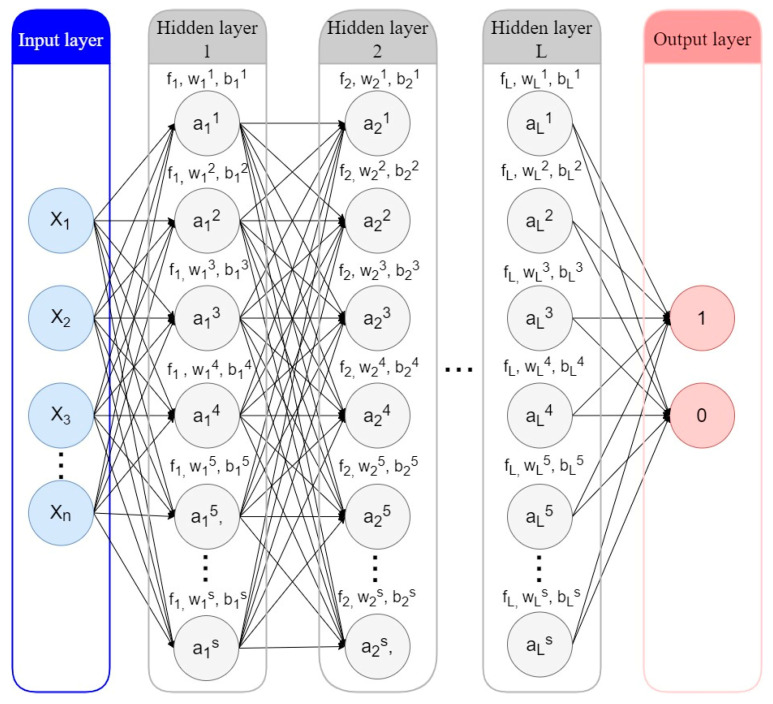
Artificial Neural Network.

ANNs, or neural networks for short, are computational models based on biological neural networks, which make up the brains of animals or humans. ANNs combine multiple processing elements based on predefined activation functions that receive inputs and generate outputs. This process is shown in Figure 10.

The neural network has n input features and L hidden layers. Each of the hidden or output nodes is calculated by Equation (5), where $f_{l}$ is the activation function (usually a non-linear function) of the *l*th layer; $w_{l}$ is the weight vector, and $b_{l}$ is the bias vector of the *l*th layer; and the input vector x is considered vector $a_{0}$.

(5) $$a_{l}=f_{l}\left(w_{l}a_{l-1}+b_{l}\right)$$

Values of the two outputs lie between 0 and 1. If the result is [1,0], it indicates a “Charged-Off” loan; on the contrary, if the result is [0,1], it indicates a “Fully-Paid” loan.

#### 5.2.5. Convolutional Neural Network (CNN)

**Figure 11:**
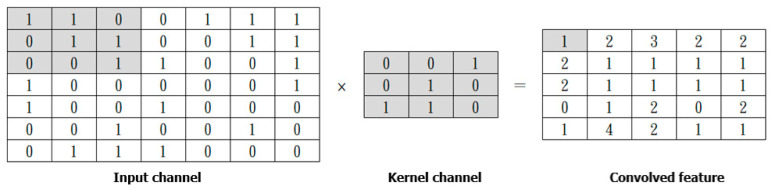
Convolutional Operation.

CNN is a deep learning algorithm, originally developed for computer vision by Y. Lecun et al. [23]. In CNN, input data are transformed into matrices (input channels), and then the input channels are filtered by the kernel channel in the Convolution Operation step. The process is shown in Figure 11.

**Figure 12:**
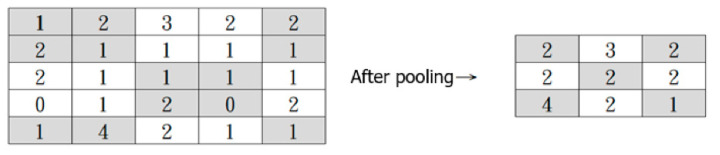
Max Pooling Operation.

The convolved feature matrix then goes through the Pooling Operation to reduce its dimension, which helps lower the computational cost and extract dominant features. There are two types of Pooling: Max Pooling and Average Pooling. The convolved feature matrix is divided into smaller parts (usually at a size of 2 × 2). In Max Pooling, the maximum values of each smaller part are returned, and in Average Pooling, the average values are returned. Figure 12 illustrates the Max Pooling Operation.

The output of the above processes is then fed into a regular neural network to solve the classification problem.

## 6. Evaluation Measures

In this section, the confusion matrix series of metrics, AUC-ROC curve, Kolmogorov–Smirnov chart (KS), and Student’s t-test are adopted to measure the efficiency of models in Section 5.

### 6.1. The Confusion Matrix Series of Metrics

The confusion matrix is used to summarize the performance of a classification algorithm. Since classification accuracy alone can be misleading, a confusion matrix can help better evaluate a model’s pros and cons. The confusion matrix is divided into four categories: true positive (TP), false positive (FP), true negative (TN), and false negative (FN). False positive (FP) and false negative (FN) correspond to Type I error and Type II error. Table 2 demonstrates the confusion matrix.

The confusion matrix elements used in this paper can be interpreted as follows:

- TP: the number of “Charged-Off” loans correctly predicted;
- FP: the number of “Charged-Off” loans incorrectly predicted (Type I error);
- TN: the number of “Fully-Paid” loans correctly predicted;
- FN: the number of “Fully-Paid” loans incorrectly predicted (Type II error).

A series of metrics are derived to evaluate the models from the above four elements in the confusion matrix. This paper adopts the ratios of specificity, negative predictive value (NPV), precision, recall, F-measure, and Kappa.

#### 6.1.1. Specificity (True Negative Rate—TNR)

Specificity measures the proportion of actual negative cases (“Fully-Paid” loans) that are correctly identified.

(6) $$\mathrm{Specificity}=\frac{TN}{TN+FP}$$

#### 6.1.2. Negative Predictive Value (NPV)

The negative predictive value (NPV) measures the proportion of predicted negative cases that are correctly identified.

(7) $$\mathrm{NPV}=\frac{TN}{TN+FN}$$

#### 6.1.3. Precision (Positive Predictive Value—PPV)

Precision measures the proportion of predicted positive cases (“Charged-Off” loans) that are correctly identified.

(8) $$\mathrm{Precision}=\frac{TP}{TP+FP}$$

#### 6.1.4. Recall (Sensitivity) 

The recall rate measures the proportion of actual positive cases that are correctly identified.

(9) $$\mathrm{Recall}=\frac{TP}{TP+FN}$$

The above metrics are relatively effective measurements for the models; however, each of them in isolation does not provide the complete picture of the performance of classifiers, especially in imbalanced datasets. Although in the data pre-processing step, this study already handled the problem of data imbalance, we still introduced two other metrics called F-measure and Kappa, as follows.

#### 6.1.5. F-Measure

It is common to summarize precision and recall as a single metric, the F-measure, which is the harmonic mean. The value of the F-measure lies between 0 and 1; 0 indicates the worst, and 1 indicates a perfect value.

(10) $$\mathrm{F-measure}=2\times \frac{Precision\times Recall}{\;Precision+Recall}$$

#### 6.1.6. Kappa (Cohen’s Kappa Coefficient)

The following formula calculates Cohen’s kappa coefficient [24]:

(11) $$\mathrm{Kappa}=\frac{p_{0}-p_{e}}{\;1-p_{e}}$$

where $p_{0}$ is the observed accuracy of the model, and $p_{e}\;$is the expected accuracy (the accuracy that any random classifier is expected to achieve based on the confusion matrix). $p_{0}$ is computed as follows:

(12) $$p_{0}=\frac{TP+TN}{TP+TN+FP+FN}$$

To calculate $p_{e}$, we need two other ratios: $p_{1}\;$and $p_{2}$.

$p_{1}$ represents the random probability that a sample in the dataset is an actual “Charged-Off” loan. From the confusion matrix, we can calculate $p_{1}$ as follows:

(13) $$p_{1}=\frac{TP+FN}{TP+FN+FP+TN}$$

(1 − $p_{1}$) is the random probability that a sample in the dataset is an actual “Fully-Paid” loan.

$p_{2}$ represents the probability that our model predicts a “Charged-Off” loan. From the confusion matrix, we can calculate $p_{2}$ as follows:

(14) $$p_{2}=\frac{TP+FP}{TP+FP+TN+FN}$$

(1 − $p_{2}$) is the probability that our model predicts a “Fully-Paid” loan.

Expected accuracy measures the agreement between our model predictions and the actual class values, which is shown in Equation (15).

(15) $$p_{e}=p_{1}\times p_{2}+\left(1-p_{1}\right)\times \left(1-p_{2}\right)\;$$

Basically, the Kappa coefficient demonstrates how much better one classifier does compared to another classifier that just guesses at random using the frequency of each class. The Kappa value ranges from −1 to +1. If the value is less than or equal to 0, the classifier is impractical. For Kappa values greater than 0, Landis et al. [25] proposed a measurement for the value: 0–0.20 as slight, 0.21–0.40 as fair, 0.41–0.60 as moderate, 0.61–0.80 as substantial, and 0.81–1 as almost perfect.

### 6.2. AUC-ROC Curve

**Figure 13:**
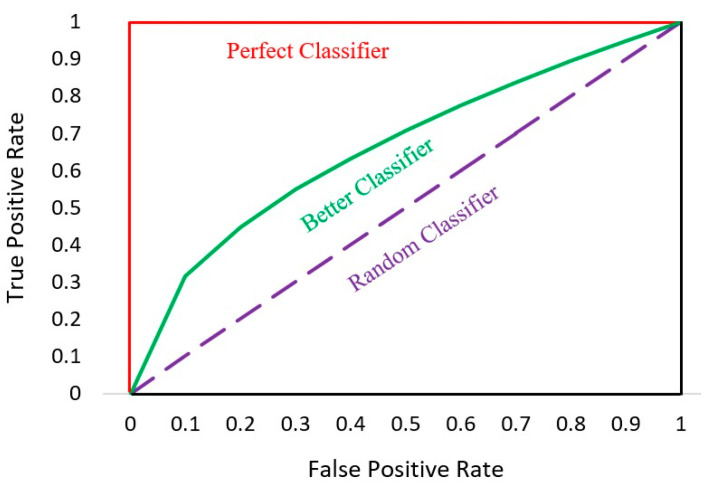
AUC-ROC curve.

The Receiver Operator Characteristic (ROC) curve is a probability curve used as an evaluation metric for binary classification problems. The ROC curve plots the true positive rate against the false positive rate, which is shown in Figure 13.

The Area Under the Curve (AUC) is defined precisely by its name; it is the area under the ROC curve. AUC measures the ability of a model to distinguish between classes and is used as a summary of the ROC curve. The higher the AUC, the better the classifier’s performance at distinguishing between “Fully-Paid” and “Charged-Off” classes.

### 6.3. Kolmogorov–Smirnov Chart (KS)

**Figure 14:**
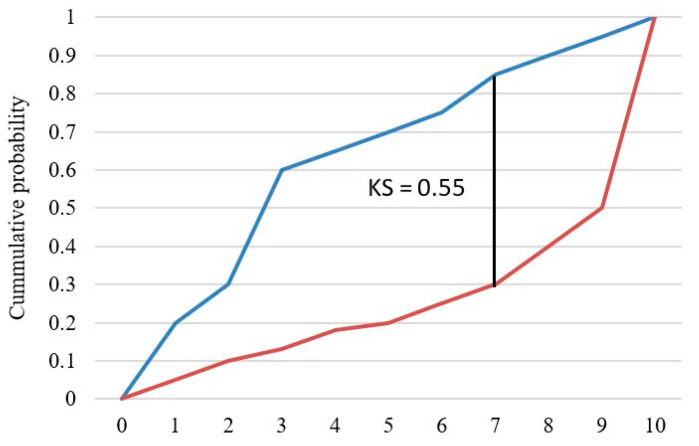
KS chart.

The KS test examines whether two random variables have the same probability distribution. In this study, the KS test looked at the maximum difference between the cumulative distribution of “Fully-Paid” loans and the cumulative distribution of “Charged-Off” loans, which is shown in Figure 14.

The blue curve represents the cumulative distribution of “Fully-Paid” loans, and the orange curve represents the cumulative distribution of “Charged-Off” loans. The KS value is the maximum difference between the two curves, and the higher the KS value, the better the model separates the two classes.

### 6.4. Student’s t-Test

This study used a two independent samples *t*-test [26] to verify the differences between each model using the above metrics. The formula is as follows:

(16) $$t=\frac{\bar{x_{1}}-\bar{x_{2}}-\mu_{0}}{\sqrt{\frac{\sum_{i=1}^{n}{\left(x_{1i}-\bar{x_{1}}\right)}^{2}+\sum_{i=1}^{n}{\left(x_{2i}-\bar{x_{2}}\right)}^{2}}{\left(n-1\right)\times n}}}$$

where

$\bar{x_{1}}$, $\bar{x_{2}}$: The means of the two samples;
$\mu_{0}$: The difference between the means of the two populations, where $\mu_{0}$ is expected to be 0;
*i* = 1, 2 … *n*;
*n*: sample size.

The null hypothesis and the alternative hypothesis are as follows:

(17) $$H_{0}\;:\bar{x_{1}}-\bar{x_{2}}=\mu_{0}$$

(18) $$H_{1}\;:\bar{x_{1}}-\bar{x_{2}}\neq \mu_{0}$$

Based on the degrees of freedom (*n* – 1) and the significance levels (0.10, 0.05, and 0.01), the t-test can reject the null hypothesis if the *p*-value is less than each significance level, indicating a significant difference between the two models.

## 7. Results Analysis

In this study, each model was ranked based on the average results of the 10-fold validation process. After that, we used Student’s t-test to determine whether each model is significantly different from the others using the evaluation metrics. This research adopted the t-test with 90%, 95%, and 99% confidence levels.

The first common-sense metric is accuracy, which summarizes the model’s performance by dividing the number of correct predictions by the total number of input samples. In terms of this measurement, empirical results from Table 3 show that LightGBM performs the best at 68.57%. The *p*-values from Table 4 also prove that LightGBM is significantly better than all other models at the 99% confidence level. The comparison in Table 5 further clarifies this difference.

As demonstrated in Table 5, other models have an average accuracy of 65.66%, which is 2.91% lower than the accuracy of LightGBM (68.57%). This number means that if LightGBM is chosen over other models, it will save 2.91% of the platform’s total debts from being mispredicted. Imagine that if we misclassify a “Fully-Paid” loan as a “Charged-Off” loan, we will lose a revenue stream from that potential customer. Conversely, if we misclassify a “Charged-Off” loan as a “Fully-Paid” loan, the lender will lose the corresponding amount from the borrower. Under the assumption that all of the loans contribute equally to LC’s revenue, a 2.91% improvement in loan classification accuracy will also improve the revenue proportionally.

According to LC’s financial reports [27], the company’s 2021 revenue was USD 818,600,000, so an improvement of 2.91% will result in a revenue increase of USD 23,821,260—an impressive number.

Besides accuracy, Table 3 also shows that LightGBM outperforms other models for most evaluation metrics, except for recall rate, where the result of the Bayesian Classifier is the highest. Similar to Table 4, the *p*-values of Student’s t-test for all other metrics are available in Appendix B. We summarize the top models under each measurement in Table 6. In terms of specificity, Student’s t-test does not demonstrate a significant difference between LightGBM and its next-ranked models—CNN and ANN. Similarly, there is no evidence that LightGBM is significantly better than the Bayesian Classifier, Logistic Regression, LDA, and ANN according to the NPV metric. The Bayesian Classifier’s recall rate significantly dominates those of other models, except for Random Forest. For the F-measure, LightGBM is also not significantly superior to the Bayesian Classifier.

This research aims to minimize the probability of default risk—the risk that occurs when actual “Charged-Off” loans are incorrectly predicted as “Fully-Paid” loans. In other words, we want to minimize false negatives (Type II error). Therefore, the key metrics in this research are accuracy, AUC value, KS value, F-measure, and Kappa. Using these metrics, Table 6 demonstrates that LightGBM dominates all other models. Other metrics, such as specificity and NPV, measure the model’s ability to identify “Fully-Paid” loans (to fix Type I error). Although this study does not focus on Type I error, these metrics also help to prevent some research bias. Overall, we recommend LightGBM as the most promising loan classification model for LC. For details of the model’s parameters, please refer to Appendix C.

## 8. Discussion and Conclusions

### 8.1. Discussion

The operation of P2P platforms is still in its development stage. When this study was proposed, many P2P platforms still had no specific legal adjustments or guarantees for personal credit. While charging handling fees to match lenders and borrowers, they offer only primary regulations to protect their customers’ rights. If there is a dispute between the two parties, it falls under civil law related to lending and borrowing. There are almost no mortgage or other guarantees for debt release, debt recovery, or similar issues. Therefore, this research aimed to develop a default prediction model to serve investors and P2P platforms to reduce default risks and information asymmetry risks so that all parties can trust each other and thus liquidate this emerging financial market.

Despite being the largest P2P lending platform in the USA, the default rate of LC, as demonstrated in Figure 2, is still very high, which proves that this platform has not been effective in debt classification. Thus, it is not difficult to recognize the similar potential risk of other smaller P2P lending companies. By solving this problem of LC in particular, and P2P lending platforms in general, our paper contributes to other subjects as well. All eight models selected to predict the default risk of LC produce relatively good results. Although the difference in accuracy between models is not too high, it is significant when applied to a sizeable P2P lending platform because “even when the default rate increases by 0.1 percent, it will cause large losses to the platform and investors” and “even a 0.1 percent improvement is significant for P2P platforms.” [11]. In addition, other financial institutions such as banks or policymakers may also consider applying AI and statistical models to solve similar problems, depending on their dataset and the techniques available. 

We adopted nine different metrics to measure the performance of the models. Except for the recall rate, all metrics prove that LightGBM almost outperforms the other models. We also carefully applied Student’s t-test to reinforce the significance of this conclusion. Therefore, we suggest that LightGBM be widely applied in credit rating in the P2P lending industry. Other financial institutions can also utilize LightGBM on their datasets to test the effectiveness of this model.

### 8.2. Conclusions

This study contributes to predicting the default risk of P2P lending platforms, both in theory and in practice.

For theoretical contributions, based on the data disclosed by Lending Club, four data pre-processing steps are proposed to improve data quality. In comparison to previous studies, we selected features by excluding post-event variables and balanced the dataset to a ratio of 1:1. We adopted eight methods to construct default prediction classifiers, including three statistical models and five AI models. These models were evaluated by the confusion matrix series of metrics, AUC-ROC curve, and KS chart, and lastly, Student’s t-test was used to examine whether there were significant differences between the models. Our study synthesizes almost all theoretically necessary steps, from data processing to building default risk prediction models and evaluation methods, the concepts of which are explained in a systematic and easy-to-understand manner. Thus, by referencing our paper, readers new to AI or statistics can grasp the fundamental knowledge and be relatively up to date with the most recently applied models.

For practical contributions, this study finds that LightGBM significantly outperforms the other models by 2.91% using the accuracy metric. For a large-scale P2P platform such as Lending Club, even a slight improvement in default prediction can significantly impact revenue. However, although Lending Club manages to collect big data from its customers, it has not adequately utilized the dataset [8]. In the Results Analysis section, we prove that if LightGBM is applied, it is expected to increase the revenue of Lending Club by nearly USD 24 million compared to other models. Besides that, if the effectiveness of information can be enhanced, the accuracy of auditing can be improved, the risk of default on the platform can be reduced, and the lender’s rights can also be indirectly protected, so lenders will be more willing to invest funds in the P2P market. Borrowers will also be more likely to participate in a healthy financial environment.

Due to the limitations of this research, there are still some issues that require further study: (1) This study does not focus on optimizing the parameters or conducting sensitivity analyses, so we recommend that future studies deploy algorithms to automate the optimization of parameters for better results. (2) The pre-processing procedures proposed in this study can be improved by optimizing the proportion of data samples. Besides the undersampling technique, future research can try oversampling or the Synthetic Minority Oversampling Technique (SMOTE). (3) Future studies may exploit feature selection methods such as genetic algorithms (GA), stepwise regression, or particle swarm optimization (PSO). (4) The same research process can be applied to other datasets in various P2P lending markets or by screening the time periods. Finally (5), we encourage further studies to use innovative models or combinations of multiple algorithms to predict P2P lending default risks.

## Figures and Tables

**Table 1:** Number of samples in “Fully-Paid” and “Charged-Off” classes.

Loan Status	Quantity	Proportion
Fully-Paid	52,117	85.45%
Charged-Off	8876	14.55%
Total	60,993	100%

**Table 2:** Confusion matrix.

	Actual “Charged-Off”	Actual “Fully-Paid”
Predicted “Charged-Off”	True Positive (TP)	False Positive (FP) Type I error
Predicted “Fully-Paid”	False Negative (FN) Type II error	True Negative (TN)

**Table 3:** Empirical results of all models.

	LightGBM	CNN	Logistic Regression	LDA	ANN	Bayesian Classifier	Random Forest	Decision Tree
Accuracy	68.57%	67.27%	66.87%	66.81%	66.85%	64.27%	63.89%	63.63%
AUC value	74.92%	73.56%	72.82%	72.76%	73.63%	68.58%	69.06%	65.59%
KS value	38.37%	35.81%	34.97%	34.90%	36.22%	30.49%	28.93%	27.93%
Specificity	71.47%	69.53%	67.32%	67.40%	69.28%	56.41%	57.56%	67.62%
NPV	67.55%	66.58%	66.73%	66.62%	66.28%	67.11%	65.92%	62.60%
Recall	65.66%	64.94%	66.44%	66.23%	64.50%	72.16%	70.23%	59.62%
Precision	69.73%	68.24%	67.05%	67.03%	67.91%	62.44%	62.33%	64.87%
F-measure	67.62%	66.43%	66.72%	66.61%	65.95%	66.82%	66.04%	62.11%
Kappa	37.13%	34.49%	33.75%	33.62%	33.75%	28.56%	27.79%	27.24%

**Table 4:** The *p*-values for model accuracy.

	LightGBM	CNN	Logistic Regression	LDA	ANN	Bayesian Classifier	Random Forest	Decision Tree
LightGBM	1.0000							
CNN	0.0034 ***	1.0000						
Logistic Regression	0.0005 ***	0.3593	1.0000					
LDA	0.0012 ***	0.3450	0.9011	1.0000				
ANN	0.0006 ***	0.3425	0.9606	0.9378	1.0000			
Bayesian Classifier	0.0000 ***	0.0000 ***	0.0000 ***	0.0001 ***	0.0000 ***	1.0000		
Random Forest	0.0000 ***	0.0000 ***	0.0000 ***	0.0000 ***	0.0000 ***	0.4524	1.0000	
Decision Tree	0.0000 ***	0.0000 ***	0.0000 ***	0.0000 ***	0.0000 ***	0.2859	0.6431	1.0000

*** Significant at the 1% level.

**Table 5:** LC’s revenue improvement based on model accuracy.

	LightGBM	CNN	Logistic Regression	LDA	ANN	Bayesian Classifier	Random Forest	Decision Tree
Accuracy	**68.57%**	67.27%	66.87%	66.81%	66.85%	64.27%	63.89%	63.63%
		**Average: 65.66%**						
Difference		**2.91%**						
LC’s revenue							USD 818,600,000	
Revenue improvement							**USD 23,821,260**	

**Table 6:** The top model(s) under each metric.

	The Best Model	Model(s) Have No Significant Difference from the Best Model
Accuracy	LightGBM	
AUC value	LightGBM	
KS value	LightGBM	
Specificity	LightGBM	CNN, ANN
NPV	LightGBM	Bayesian Classifier, Logistic Regression, LDA, ANN
Recall	Bayesian Classifier	Random Forest
Precision	LightGBM	
F-measure	LightGBM	Bayesian Classifier
Kappa	LightGBM	

## Data Availability

The datasets for this study are available upon request from the corresponding author.

## Appendix A. Detailed Description of Features

**Table A1:** Detailed description of features.

ORDER	FEATURES	DESCRIPTONS
1	acc_open_past_24mths	Number of trades opened in past 24 months.
2	annual_inc	The self-reported annual income provided by the borrower during registration.
3	application_type	Indicates whether the loan is an individual application or a joint application with two co-borrowers.
4	chargeoff_within_12_mths	Number of Charged-Offs within 12 months.
5	collections_12_mths_ex_med	Number of collections in 12 months excluding medical collections.
6	delinq_2yrs	The number of 30+ days past-due incidences of delinquency in the borrower’s credit file for the past 2 years.
7	delinq_amnt	Accounts on which the borrower is now delinquent.
8	fico_range_high	The upper boundary range that the borrower’s FICO at loan origination belongs to.
9	fico_range_low	The lower boundary range that the borrower’s FICO at loan origination belongs to.
10	funded_amnt	The total amount committed to that loan at that point in time.
11	funded_amnt_inv	The total amount committed by investors to that loan at that point in time.
12	grade	LC-assigned loan grade.
13	home_ownership	The home ownership status provided by the borrower during registration or obtained from the credit report. Our values are: RENT, OWN, MORTGAGE, and OTHER.
14	initial_list_status	The initial listing status of the loan. Possible values are—W, F.
15	inq_fi	Number of personal finance inquiries.
16	inq_last_12m	Number of credit inquiries in past 12 months.
17	inq_last_6mths	The number of inquiries in past 6 months (excluding auto and mortgage inquiries).
18	installment	The monthly payment owed by the borrower if the loan originates.
19	int_rate	Interest rate on the loan.
20	issue_d	The month in which the loan was funded.
21	loan_amnt	The listed amount of the loan applied for by the borrower. If at some point in time, the credit department reduces the loan amount, then it will be reflected in this value.
22	max_bal_bc	Maximum current balance owed on all revolving accounts.
23	mo_sin_old_rev_tl_op	Months since oldest revolving account opened.
24	mo_sin_rcnt_rev_tl_op	Months since most recent revolving account opened.
25	mo_sin_rcnt_tl	Months since most recent account opened.
26	mort_acc	Number of mortgage accounts.
27	num_accts_ever_120_pd	Number of accounts ever 120 or more days past due.
28	num_actv_bc_tl	Number of currently active bankcard accounts.
29	num_actv_rev_tl	Number of currently active revolving trades.
30	num_bc_sats	Number of satisfactory bankcard accounts.
31	num_bc_tl	Number of bankcard accounts.
32	num_il_tl	Number of installment accounts.
33	num_op_rev_tl	Number of open revolving accounts.
34	num_rev_accts	Number of revolving accounts.
35	num_rev_tl_bal_gt_0	Number of revolving trades with balance >0.
36	num_sats	Number of satisfactory accounts.
37	num_tl_90g_dpd_24m	Number of accounts 90 or more days past due in last 24 months.
38	num_tl_op_past_12m	Number of accounts opened in past 12 months.
39	open_acc	The number of open credit lines in the borrower’s credit file.
40	open_acc_6m	Number of open trades in last 6 months.
41	open_act_il	Number of currently active installment trades.
42	open_il_12m	Number of installment accounts opened in past 12 months.
43	open_il_24m	Number of installment accounts opened in past 24 months.
44	open_rv_12m	Number of revolving trades opened in past 12 months.
45	open_rv_24m	Number of revolving trades opened in past 24 months.
46	pct_tl_nvr_dlq	Percent of trades never delinquent.
47	pub_rec	Number of derogatory public records.
48	pub_rec_bankruptcies	Number of public record bankruptcies.
49	purpose	A category provided by the borrower for the loan request.
50	revol_bal	Total credit revolving balance.
51	term	The number of payments on the loan. Values are in months and can be either 36 or 60.
52	title	The loan title provided by the borrower.
53	tot_coll_amt	Total collection amounts ever owed.
54	tot_cur_bal	Total current balance of all accounts.
55	tot_hi_cred_lim	Total high credit/credit limit.
56	total_acc	The total number of credit lines currently in the borrower’s credit file.
57	total_bal_ex_mort	Total credit balance excluding mortgage.
58	total_bal_il	Total current balance of all installment accounts.
59	total_bc_limit	Total bankcard high credit/credit limit.
60	total_cu_tl	Number of finance trades.
61	total_il_high_credit_limit	Total installment high credit/credit limit.
62	total_rev_hi_lim	Total revolving high credit/credit limit.
63	verification_status	Indicates if income was verified by LC, not verified, or if the income source was verified.

## Appendix B. Model’s p-Values for Each Metric

**Table A2:** The *p*-values for AUC metric.

	Bayesian Classifier	CNN	Random Forest	LDA	LightGBM	Logistic Regression	ANN	Decision Tree
Bayesian Classifier	1.0000							
CNN	0.0000 ***	1.0000						
Random Forest	0.2521	0.0000 ***	1.0000					
LDA	0.0000 ***	0.1008	0.0000 ***	1.0000				
LightGBM	0.0000 ***	0.0110 **	0.0000 ***	0.0001 ***	1.0000			
Logistic Regression	0.0000 ***	0.1115	0.0000 ***	0.8963	0.0001 ***	1.0000		
ANN	0.0000 ***	0.8995	0.0000 ***	0.0627 *	0.0103 **	0.0684 *	1.0000	
Decision Tree	0.0000 ***	0.0000 ***	0.0000 ***	0.0000 ***	0.0000 ***	0.0000 ***	0.0000 ***	1.0000

* Significant at the 10% level; ** significant at the 5% level; *** significant at the 1% level.

**Table A3:** The *p*-value for KS metric.

	Bayesian Classifier	CNN	Random Forest	LDA	LightGBM	Logistic Regression	ANN	Decision Tree
Bayesian Classifier	1.0000							
CNN	0.0000 ***	1.0000						
Random Forest	0.0596 *	0.0000 ***	1.0000					
LDA	0.0000 ***	0.2967	0.0000 ***	1.0000				
LightGBM	0.0000 ***	0.0046 ***	0.0000 ***	0.0002 ***	1.0000			
Logistic Regression	0.0000 ***	0.3013	0.0000 ***	0.9358	0.0001 ***	1.0000		
ANN	0.0000 ***	0.6565	0.0000 ***	0.1491	0.0175 **	0.1459	1.0000	
Decision Tree	0.0176 **	0.0000 ***	0.3528	0.0000 ***	0.0000 ***	0.0000 ***	0.0000 ***	1.0000

* Significant at the 10% level; ** significant at the 5% level; *** significant at the 1% level.

**Table A4:** The *p*-value for specificity metric.

	Bayesian Classifier	CNN	Random Forest	LDA	LightGBM	Logistic Regression	ANN	Decision Tree
Bayesian Classifier	1.0000							
CNN	0.0000 ***	1.0000						
Random Forest	0.5197	0.0000 ***	1.0000					
LDA	0.0000 ***	0.2096	0.0000 ***	1.0000				
LightGBM	0.0000 ***	0.2349	0.0000 ***	0.0002 ***	1.0000			
Logistic Regression	0.0000 ***	0.1868	0.0000 ***	0.9341	0.0001 ***	1.0000		
ANN	0.0000 ***	0.9130	0.0000 ***	0.3059	0.2236	0.2803	1.0000	
Decision Tree	0.0000 ***	0.2980	0.0000 ***	0.8565	0.0027 ***	0.8007	0.3979	1.0000

*** Significant at the 1% level.

**Table A5:** The *p*-value for NPV.

	Bayesian Classifier	CNN	Random Forest	LDA	LightGBM	Logistic Regression	ANN	Decision Tree
Bayesian Classifier	1.0000							
CNN	0.4795	1.0000						
Random Forest	0.1473	0.3456	1.0000					
LDA	0.5267	0.9475	0.3309	1.0000				
LightGBM	0.5254	0.0974 *	0.0169 **	0.1282	1.0000			
Logistic Regression	0.6185	0.8117	0.2553	0.8688	0.1677	1.0000		
ANN	0.4093	0.7425	0.7113	0.7125	0.1555	0.6241	1.0000	
Decision Tree	0.0000 ***	0.0000 ***	0.0002 ***	0.0000 ***	0.0000 ***	0.0000 ***	0.0010 ***	1.0000

* Significant at the 10% level; ** significant at the 5% level; *** significant at the 1% level.

**Table A6:** The *p*-value for recall rate.

	Bayesian Classifier	CNN	Random Forest	LDA	LightGBM	Logistic Regression	ANN	Decision Tree
Bayesian Classifier	1.0000							
CNN	0.0029 ***	1.0000						
Random Forest	0.2565	0.0023 ***	1.0000					
LDA	0.0021 ***	0.3997	0.0000 ***	1.0000				
LightGBM	0.0008 ***	0.6293	0.0000 ***	0.4186	1.0000			
Logistic Regression	0.0026 ***	0.3261	0.0001 ***	0.7801	0.2542	1.0000		
ANN	0.0045 ***	0.8472	0.0062 ***	0.3614	0.5326	0.3057	1.0000	
Decision Tree	0.0000 ***	0.0027 ***	0.0000 ***	0.0000 ***	0.0000 ***	0.0000 ***	0.0182 **	1.0000

** Significant at the 5% level; *** significant at the 1% level.

**Table A7:** The *p*-value for precision.

	Bayesian Classifier	CNN	Random Forest	LDA	LightGBM	Logistic Regression	ANN	Decision Tree
Bayesian Classifier	1.0000							
CNN	0.0000 ***	1.0000						
Random Forest	0.8778	0.0000 ***	1.0000					
LDA	0.0000 ***	0.1339	0.0000 ***	1.0000				
LightGBM	0.0000 ***	0.0469 **	0.0000 ***	0.0003 ***	1.0000			
Logistic Regression	0.0000 ***	0.1155	0.0000 ***	0.9848	0.0001 ***	1.0000		
ANN	0.0000 ***	0.7368	0.0000 ***	0.3504	0.0467 **	0.3366	1.0000	
Decision Tree	0.0089 ***	0.0006 ***	0.0011 ***	0.0083 ***	0.0000 ***	0.0050 ***	0.0048 ***	1.0000

** Significant at the 5% level; *** significant at the 1% level.

**Table A8:** The *p*-value for F-measure.

	Bayesian Classifier	CNN	Random Forest	LDA	LightGBM	Logistic Regression	ANN	Decision Tree
Bayesian Classifier	1.0000							
CNN	0.6439	1.0000						
Random Forest	0.2736	0.5760	1.0000					
LDA	0.7704	0.7901	0.2724	1.0000				
LightGBM	0.2350	0.0812 *	0.0025 ***	0.0395 **	1.0000			
Logistic Regression	0.8865	0.6531	0.1487	0.8115	0.0347 **	1.0000		
ANN	0.3506	0.6048	0.9170	0.4036	0.0354 **	0.3093	1.0000	
Decision Tree	0.0000 ***	0.0000 ***	0.0000 ***	0.0000 ***	0.0000 ***	0.0000 ***	0.0001 ***	1.0000

* Significant at the 10% level; ** significant at the 5% level; *** significant at the 1% level.

**Table A9:** The *p*-value for Kappa.

	Bayesian Classifier	CNN	Random Forest	LDA	LightGBM	Logistic Regression	ANN	Decision Tree
Bayesian Classifier	1.0000							
CNN	0.0000 ***	1.0000						
Random Forest	0.4293	0.0000 ***	1.0000					
LDA	0.0001 ***	0.3739	0.0000 ***	1.0000				
LightGBM	0.0000 ***	0.0029 ***	0.0000 ***	0.0011 ***	1.0000			
Logistic Regression	0.0000 ***	0.3938	0.0000 ***	0.8992	0.0005 ***	1.0000		
ANN	0.0000 ***	0.4040	0.0000 ***	0.8964	0.0006 ***	0.9956	1.0000	
Decision Tree	0.2596	0.0000 ***	0.6309	0.0000 ***	0.0000 ***	0.0000 ***	0.0000 ***	1.0000

*** Significant at the 1% level.

## Appendix C. AI Model’s Parameters

Parameters for statistical models are not considered. Table A10 shows the parameters of AI models used in this study.

**Table A10:** AI model’s parameters.

MODEL	PARAMETERS	VALUES
**Decision Tree**	Criterion	‘gini’
	Splitter	‘best’
	Min_samples_leaf	1
**RandomForest**	Criterion	entropy
	N_estimators	10
	N_jobs	1
**CNN**	Conv1D[filters, kernel_size, activation, input_shape]	[64, 3, sigmoid, 63]
	MaxPooling1D	2
	Conv1D[filters, kernel_size, activation]	[64, 3, sigmoid]
	MaxPooling1D	2
	Conv1D[filters, kernel_size, activation]	[64, 3, sigmoid]
	Flatten	
	Dense[units, activation]	[64, sigmoid]
	Dense[units, activation]	[1, sigmoid]
	Optimizer	adam
	Loss	binary_crossentropy
	Epochs	1000
	Batch_size	2000
**ANN**	Dense[units, activation]	[63, sigmoid]
	Dense[units, activation]	[25, sigmoid]
	Dense[units, activation]	[50, sigmoid]
	Dense[units, activation]	[100, sigmoid]
	Dense[units, activation]	[50, sigmoid]
	Dense[units, activation]	[25, sigmoid]
	Dense[units, activation]	[1, sigmoid]
	Optimizer	adam
	Loss	binary_crossentropy
	Epochs	5000
	Batch_size	2000
**LightGBM**	Learning_rate	0.01
	Max_depth	−1
	Num_iteration	300
	Objective	binary
